# With the Sick Children, Great Ormond-Street

**Published:** 1889-05-25

**Authors:** 


### The Hospital Workshop.

### I.—WITH THE SICK CHILDREN, GREAT ORMOND STREET.

(BY OUR SPECIAL COMMISSIONER.)

A SAD sight, that of suffering children ; yet it is comforting to know withal there are places whither they may be taken in the full assurance of obtaining the best treatment that modern science can afford. The out-patient department at Great Ormond-street is well arranged. There are plenty of seats in the large and comfortable waiting-room, where warmth, light, and ventilation are alike good. Cakes and milk can be purchased from a woman who attends for the purpose of providing them. A sister is in charge. Patients are admitted at 8.30 a.m., and the porter gives to each one on arrival a metal ticket with a number, which settles the order of admission to the consulting-room. These things are mentioned here because they are by no means to be met with in all the institutions which it is the purpose of these sketches to describe from time to time. Further points of importance, from the patient's point of view, are (1) members of the staff are punctual in their attendance, and (2) there is no delay about dispensing the medicines.

The out-patients average 1,400 a week, and children brought for treatment must not be over twelve years of age. One friend or relative is allowed in the consulting-room with each little applicant. A subscriber's letter is asked for, but at the same time no one is sent away or refused treatment for want of such a recommendation. Pay patients are not admitted to this hospital, which professes to treat poor people only, and the following rule is printed in the regulations :

"No child whose parent is in receipt of more than 40s. per week shall, as a rule, be considered eligible for treatment as an out-patient of the hospital, and all patients who do not bring a letter from a governor or subscriber will be required to make a statement as to the circumstances of the family, and submit to have such statement investigated."

When any doubt occurs, the case is forwarded to the Charity Organisation Society. It is a significant fact that years ago, when a systematic enquiry was made into the means of applicants, a considerable falling off in attendance was the result. Be the explanation what it may, the visitor will soon see that the abject poor form a small proportion of those present in the out-patient room, an observation which applies to most of the London hospitals, with the exception of a few at the East End. Many of the Ormond-street patients live in the immediate neighbourhood, especially about Drury-lane, and the Italian colony in Leather-lane ; others hail from King's Cross, Islington, and different parts of the town ; while the remainder come from varying distances in the country.

On entering the consulting-room, the patients are seen by the physician or the surgeon of the day, aided by one or more clinical assistants, who must be qualified men. There are generally a few students and lady doctors present, to whom the practice of the hospital is open on payment of a small fee.

Having thus outlined the working of the department, let us take a peep—there is not time for more—into the surgeon's consulting-room. This first case seems rather more fitted for the medical side ; but there is most likely some good reason for its appearance here—perhaps a history of a blow or other injury. A little girl of three is lying in her mother's arms with the flushed face and languid look suggestive of a febrile attack. The eyes are closed, the nostrils dilated, the breathing quick. She puts out her tongue when asked to do so, but without opening her eyes or moving otherwise. Upon trial, the thermometer shows a temperature of 102°·1, and with the stethoscope "crepitations" can be heard at the bases of both lungs. Clearly a case for prompt admission, and not unlikely one where skilled nursing will be more than half the battle. Accordingly, the little patient is sent on to the wards.

Here is a boy of five, whose legs are bent outwards to such an extent that one might pass a fair-sized football between them without touching either knee. A glance tells one this is due to rickets, a terribly common disease here, almost every second or third child having some trace or other of that condition. Dr. Cheadle, the senior physician to the hospital, has lately shown that rickets depends almost solely on improper feeding during infancy. In his interesting lectures on the subject he mentions the fact that the young lions at the Zoological Gardens grow up "rickety" or soft-boned when fed on farinaceous food, but that this does not occur upon a diet of flesh or pounded bones. There can be no doubt that hitherto one of the chief causes of infant mortality has been the feeding of children under seven months of age upon starchy foods, such as arrowroot, cornflour, biscuits, and other things they are quite unable to digest. Material of this kind is so much foreign matter introduced into the stomach and intestines of the unhappy little ones. The irritation of its presence sets up diarrhœa and reflex actions of the nervous system (convulsions), along with peevishness and wasting. In fact, a baby may be practically starved by the mistaken kindness which crams gruel, arrowroot, and other unfit food into its interior. The explanation lies in a nutshell ; before the age of six months the glands for digesting starchy matter are not developed. Already the recognition and the spread of knowledge regarding these simple facts have reduced the lists of infantile mortality, and it is not too much to say that the subject is one of national importance. Furthermore, the community is benefited by being relieved of the burden of stunted, decrepit, and, perhaps, criminal individuals who represent the survivors of the terrible ordeal of unwise baby feeding.

Here is an anxious mother with a stout, cherry-cheeked youngster of six in a sailor's suit. Her tale is that the boy broke his thigh twelve months ago, and has never been the same since. "He walks fearfully ; knocks his knees together, and throws his feet out." She is also persuaded that something is wrong with the arch of the foot. On examination, it turns out to be a simple case of knock-knee. The broken bone of the thigh has been so well united that one can hardly make out where the fracture has been. As to the foot, it is quite as it should be ; so the mother—a nervous, fidgety woman, full of everyone's theories—is assured that what she imagines to be a deformity is an artistic beauty. Later on something can be done with the legs, for nowadays orthopædic surgery works wonders in the way of straightening up crooked limbs.

The avowed objects of the institution are:

I. The medical and surgical treatment of poor children.

II. The attainment and diffusion of knowledge regarding the diseases of children.

III. The training of nurses for children.

A further great and good work is done in teaching mothers how to feed their children. With this end in view many hundreds of leaflets, containing full printed directions, are distributed among the patients' friends. In the hope that other institutions of the kind may be induced to follow so excellent an example, copies are here given:

How to bring up Children.

1. Keep them warm. Let the clothing be warm (*i.e.*, flannel or woollen), but not tight. Children should wear stockings nearly up to the knees, and frocks well up to the neck, with long sleeves. Give them plenty of fresh air; take them out whenever the weather is fine. Wash the child all over with warm water daily. If possible let the child sleep in a cot by itself. Open the windows at least twice a day.

Food.

2. If the mother has plenty of breast milk, the child should not have any other food whatever, until it is seven months old. It must be suckled every two hours during the first month, and the interval gradually increased, so that at the end of three months the child is suckled every three hours. Too frequent suckling is the common cause of the sickness of infants.

3. If the mother has only a little milk, let the child have it; and in addition milk mixed as directed in Rule 4. Except when ordered by a doctor, do not on any account give the child any baked flour, arrowroot, cornflour, biscuits, tops and bottoms, or any so-called "infant's food," before it is seven months old. It is a good plan, if the mother has only a little breast milk, that she should drink a cupful of cow's milk half an hour before suckling.

4. If the child must be brought up entirely by hand, it should be fed out of a bottle with cow's milk warmed and diluted. Fresh cow's milk must be used. During the first month it must be diluted with at least twice as much water or, better still, with limewater or barley-water* in the same proportion, if the milk be of good quality; the addition of a table-spoonful of cream to each bottle when it can be obtained would render the mixture more nutritious. A small quantity of white sugar must be added to each bottle. As the child gets older, the proportion of milk must be gradually increased. The milk should always be boiled before being used. This is especially important in the case of infants under three months old, and in summer.

5. If cow's milk cannnot be digested after a fair trial, condensed milk may be given—one teaspoonful to four or five tablespoonfuls of water which has been boiled, but after a month another attempt should be made with fresh cow's milk. The best kind of bottle is the old-fashioned straight one, with an indiarubber teat, and without any tube at all. It is quite impossible to prevent corks and tubes from becoming foul and turning the milk sour. When the child has finished its meal the bottle must be rinsed, and placed in clean water until again required. Once a day it should be carefully cleaned with hot water in which a little soda has been dissolved, and then rinsed with water and a few tea leaves. On no account must the milk that remains, after the child has finished, be used again. A fresh quantity must be prepared for each time of feeding.

6. When the child has reached the age of seven months it may have one or two meals a day of milk thickened with one or other of the following malted foods—viz., Allen and Hanbury's, Mellin's, Savory and Moore's, and Squire's. Or Chapman's entire wheaten flour may be used: to prepare this, make a teaspoonful of the flour into a paste with cold water, pour a half a pint of boiling water on it, and let it simmer for twenty minutes, constantly stirring; then add it to the milk, which must be warmed. The other meals should be of milk only. The amount of these foods used may be increased as the child grows older. As a rule, the child should be gradually weaned at ten months old. At eight months the child may have broth or beef tea, in addition to the milk. At twelve months, give the yelk of an egg, lightly boiled or beaten up, or a little milk pudding. At eighteen months, give a little meat every day, scraped or pounded into soft pulp, or a little mashed potato with gravy. Cheese, beer, or spirits should never be given to children. It is a great mistake to give children under two years old "just what the parents have." It is a great mistake to give them tea instead of milk. On no account give babies teething powders and soothing syrups.

* Barley-water is prepared as follows: Two teaspoonfuls of pearl barley to a pint of cold water, boiled down to two-thirds of a pint, and then strained through muslin. A fresh quantity must be prepared at least twice a day.

Directions for the Cure of Rickety Children.

Your child is suffering from "rickets," a disease which shows its effects principally in the bones of the limbs, but which, nevertheless, affects all the other parts of the body. It results mainly from want of proper nourishment.

To improve the child's condition:

1. The diet must consist of milk and things made with milk (porridge, milk puddings, bread and milk, etc.). This must be given at regular times; nothing between meals.

2. The legs must be bound together with flannel, so as to prevent the child from walking or standing. They should be released at night, and the body bathed with tepid water in which some sea-salt is mixed.

3. The child should not be clothed too warmly by day or night.

4. The room in which it sleeps must be kept fresh and cool. The child must be taken out as much as possible.

